# Artificial MiRNA Knockdown of Platelet Glycoprotein lbα: A Tool for Platelet Gene Silencing

**DOI:** 10.1371/journal.pone.0132899

**Published:** 2015-07-15

**Authors:** Tim Thijs, Katleen Broos, Stefaan J. Soenen, Aline Vandenbulcke, Karen Vanhoorelbeke, Hans Deckmyn, Isabelle I. Salles-Crawley

**Affiliations:** 1 Laboratory for Thrombosis Research, Interdisciplinary Research Facility Life Sciences, KU Leuven Kulak, Kortrijk, Belgium; 2 Biomedical MRI, Department of Imaging and technology, KU Leuven, Leuven, Belgium; 3 Centre for Haematology, Imperial College London, London, United Kingdom; Rutgers - New Jersey Medical School, UNITED STATES

## Abstract

In recent years, candidate genes and proteins implicated in platelet function have been identified by various genomic approaches. To elucidate their exact role, we aimed to develop a method to apply miRNA interference in platelet progenitor cells by using GPIbα as a proof-of-concept target protein. After *in silico* and *in vitro* screening of siRNAs targeting GPIbα (si*GPIBA*s), we developed artificial miRNAs (mi*GPIBA*s), which were tested in CHO cells stably expressing GPIb-IX complex and megakaryoblastic DAMI cells. Introduction of si*GPIBA*s in CHO GPIb-IX cells resulted in 44 to 75% and up to 80% knockdown of GPIbα expression using single or combined siRNAs, respectively. Conversion of si*GPIBAs* to mi*GPIBA*s resulted in reduced silencing efficiency, which could however be circumvented by tandem integration of two hairpins targeting different regions of *GPIBA* mRNA where 72% GPIbα knockdown was achieved. CHO GPIb-IX cells transfected with the mi*GPIBA* construct displayed a significant decrease in their ability to aggregate characterized by lower aggregate numbers and size compared to control CHO GPIb-IX cells. More importantly, we successfully silenced GPIbα in differentiating megakaryoblastic DAMI cells that exhibited morphological changes associated with actin organization. In conclusion, we here report the successful use of miRNA technology to silence a platelet protein in megakaryoblastic cells and demonstrate its usefulness in functional assays. Hence, we believe that artificial miRNAs are suitable tools to unravel the role of a protein of interest in stem cells, megakaryocytes and platelets, thereby expanding their application to novel fields of basic and translational research.

## Introduction

Platelets play a pivotal role in thrombosis and haemostasis but also in inflammatory processes such as atherosclerosis or infectious diseases [1]. To further expand our understanding of platelets, several genomic, transcriptomic and proteomic studies have been performed leading to the identification of thousands of candidate genes for which the vast majority of them are of unknown function [2–4]. Gene silencing by RNA interference is a powerful approach to determine the function of a gene, however this cannot be applied directly to platelets as they are anucleated cells. Direct introduction of small interfering RNAs (siRNAs) in platelets is further hampered by low transfection efficiency and the high sensitivity of platelets to permeabilisation techniques, resulting in an altered physiology [5]. The marginal synthesis of proteins by platelets furthermore implies that a post-transcriptional technique such as RNA interference will only have limited success when applied directly [6]. The study of platelets in which expression of a protein is suppressed therefore requires stable genetic modification of either the megakaryocyte (progenitor of platelets) or hematopoietic stem and progenitor cells (HSPC), from which transgenic human platelets can be generated [7].

RNA interference can be achieved by introducing siRNAs directly into target cells or be produced by longer RNA precursors such as short hairpin RNAs (shRNAs) or micro RNAs (miRNAs) [8]. Although shRNA molecules have frequently been used to knock down expression of a gene of interest in various cell types, a growing number of reports have shown cytotoxic effects and immune responses triggered by shRNAs [9–12]. In light of these reports, artificial miRNA sequences, in which the stem sequence of a natural miRNA has been replaced by a sequence targeting the gene of interest represent a superior tool for efficient gene knockdown [12, 13]. In addition, as opposed to polymerase type III promoter driven shRNAs, miRNAs can be transcribed from polymerase type II promoters, which can allow targeting gene silencing to a particular cell type [12]. There are only few examples of the use of shRNA technology to genetically modify platelets via transduction of mouse or human HSPC, reviewed elsewhere [7], [10, 14–16].

The aim of our study is therefore to establish miRNA as a powerful tool to genetically modify platelets or megakaryocytic cell lines to use in platelet functional assays. As proof of principle, we developed a miRNA-expressing vector targeting GPIbα, the most functionally important subunit of the GPIb-V-IX complex. Absence or dysfunction of GPIb-V-IX results in the Bernard-Soulier Syndrome, a bleeding disorder characterised not only by impaired platelet adhesion, but also by macrothrombocytopenia, due to a disturbed link between the GPIb-V-IX complex and the underlying cytoskeleton during platelet and/or MK formation [17].

We here report the use of miRNA-expressing vectors generated by incorporation of *in vitro* validated siRNA duplexes into a human miRNA-30a (miR30) scaffold to successfully knockdown a platelet gene (GPIbα) in two cell line models. We demonstrate that cells transfected with miRNA vectors lose their ability to fully aggregate and display impaired actin cytoskeleton rearrangement.

## Materials & Methods

### Cell culture

Chinese hamster ovary (CHO) cells expressing GPIbα, GPIbβ and GPIX on their surface (CHO GPIb-IX) or CHO cells expressing only GPIbβ and GPIX and not GPIbα (CHO β9) (both kind gifts from J.A. Lopez, Puget Sound Blood Center, Seattle, WA) were cultured in α Minimum Essential Medium (Life Technologies, Carlsbad, CA) supplemented with 10% Fetal Calf Serum, 1% Penicillin-Streptomycin and in the presence of G418 (Roche, Indianapolis, IN) and/or methotrexate (Sigma-Aldrich, St. Louis, MO) as previously described [18]. Human megakaryoblastic DAMI cells were obtained from ATCC (Manassas, VA) and grown in RPMI1640 medium supplemented with 10% Fetal Calf Serum, 1% Penicillin-Streptomycin, 1% MEM NEAA and 1% sodium-pyruvate (all from Life Technologies) at 37°C and 5% CO_2_. For differentiation experiments, 1μM PMA (Merck, Darmstadt, Germany) was added to DAMI growth medium (hereafter referred to as differentiation medium).

### siRNA selection and miRNA construction

After consultation of online designer tools from Life Technologies, Dharmacon and Ambion and offline scoring according to known criteria [19, 20], three siRNA sequences were selected targeted to the *GPIBA* transcript encoding GPIbα: si*GPIBA*-1, si*GPIBA*-2, si*GPIBA*-3, respectively targeting the *GPIBA* mRNA starting at nucleotide 572, 660 and 852. Conversion of siRNA duplexes to 22nt long miRNA was done by determining which nucleotides were best omitted, based on scoring to the aforementioned criteria for siRNAs, and including a mismatch at position 1 (Table 1). This resulted in the creation of mi*GPIBA*-2 and mi*GPIBA*-3 with predicted secondary structures as depicted in Figure A in S1 File. Integration of these miRNAs in a human miR30 scaffold to generate precursor miRNAs (pre-miRNAs) and construction of miRNA vectors was done according to Paddison *et al*. [21]. The DNA template for mi*GPIBA*-2 was engineered as follow: 5’-**TGCTGTTGACAGTGAGCG**- CGAGAACTCGCTGTATACAATA (sense miRNA)-*TAGTGAAGCCACAGATGTA*- TATTGTATACAGCGAGTTCTCT (antisense miRNA)-**TGCCTACTGCCTCGGA**-3’ (bold: flanking sequence, italic: loop sequence, underscored: miRNA sequence) (Figure A in S1 File; Table 1). This template was amplified by PCR using 5’-AAAAAAGATCTCGAGATCCAAGAAGGTATATTGCTGTTGACAGTGAGCG-3’ and 5’-AAAAAGGATCCAAGTCGACATCGTAGCCCTTGAAGTCCGAGGCAGTAGGCA-3’ as forward and reverse primers, respectively. Following restriction digest, the PCR product was integrated in a pCMV-eGFP vector (Clontech, Mountain View, CA) to generate the miRNA vectors pCMV-mi*GPIBA*-2-eGFP, pCMV-eGFP-mi*GPIBA*-2, pCMV-mi*GPIBA*-3-eGFP, pCMV-mi*GPIBA*-2+2-eGFP and pCMV-mi*GPIBA*-2+3-eGFP (Figure B in S1 File). Non-targeting miRNA vectors pCMV-mi*GPIBA*-2NS-eGFP and pCMV-mi*GPIBA*-3NS-eGFP served as controls and were generated by mutating nucleotides 10–14 from sense and antisense miRNA to their reverse complement, as this region is important for target mRNA cleavage [22, 23]. The resulting sense miRNA target sequences for pCMV-mi*GPIBA*-2NS-eGFP and pCMV-mi*GPIBA*-3NS-eGFP were (5’-CGAGAACTCGGACAATACAAT-3’) and (5’-CGTGCAGTGTCTGTATTCAGAC-3’), respectively.

**Table 1:** Overview of *GPIBA* mRNA sequences targeted by siRNA and miRNA.

*GPIBA* mRNA	siRNA	miRNA
572 5’-GGAGAAGTCATCTGGCTAACAA-3’	**si*GPIBA*-1**	**mi*GPIBA*-1**
	5’-UGGAGAAGUCAUCUGGCUAACAA-3’	Not constructed
	3’-ACCUCUUCAGUAGACCGAUUGUU-5’	
660 5’-CAAGAGAACTCGCTGTATACAATA-3’	**si*GPIBA*-2**	**mi*GPIBA*-2**
	5’-CCAAGAGAACUCGCUGUAUACAAUA-3’	5’- CGAGAACTCGCTGTATACAATA-3’
	3’-GGUUCUCUUGAGCGACAUAUGUUAU-5’	3’-TCTCTTGAGCGACATATGTTAT-5’
852 5’-GGCCAGTGTGCAGTGTGACAATTCA-3’	**si*GPIBA*-3**	**mi*GPIBA*-3**
	5’-GGCCAGUGUGCAGUGUGACAAUUCA-3’	5’-CGTGCAGTGTGACAATTCAGAC-3’
	3’-CCGGUCACACGUCACACUGUUAAGU-5’	3’-ACACGTCACACTGTTAAGTCAG-5’

### Determination of siRNA and miRNA mediated knockdown in CHO GPIb-IX cells

CHO GPIb-IX cells at 90% confluence were transfected in T-25 flasks with 240 pmol siRNA oligos or 240 pmol Block IT fluorescent oligo (Life Technologies) to determine siRNA transfection efficiency or alternatively 18 μg pCMV-mi*GPIBA*-eGFP or 18 μg pCMV-eGFP using Lipofectamine2000 (Life Technologies) according to the manufacturer’s instructions. Transfection efficiencies for siRNA and miRNA mediated knockdown was assessed using Block IT fluorescent oligos (Life Technologies) and pCMV-eGFP plasmid, respectively. As previously shown [24–26], insertion of miRNA sequences targeted to a transgene in 5’ or 3’ of the ORF of a reporter gene resulted in a nearly complete loss of eGFP expression (Figure A in S2 File).

Cells were stained 48h post transfection using the anti-GPIbα monoclonal antibody (moAb) 6B4 and a goat anti-mouse-PE secondary Ab (Jackson Immunoresearch, West Grove, PA) [27]. After fixation, cells were analyzed on an EPICS XL-MCL Flow Cytometer (Beckman-Coulter, Fullerton, CA). A gate was set for viable CHO GPIb-IX cells as determined by propidium iodide staining (data not shown) where 10,000 events were collected. Analysis was done using Flowing software 2.5 version (http://www.flowingsoftware.com/).

### CHO GPIb-IX aggregation assay

The CHO GPIb-IX cell aggregation assay was performed as previously described with minor modifications [28]. Cells grown at 90–95% confluence in a 12-well plate were transfected in Optimem (Invitrogen) using per well a mixture of 1.5 μg pDNA and 3.71 μg polyethyleneimine (Polysciences, Warrington, PA) resuspended in 150mM NaCl, which had been pre-incubated for 20 min prior to transfection. After 24h, transfection solution was replaced with culture medium for another 24h after which harvested cells (2x10^5^ cells) were transferred to a 24-well plate and incubated with 2.5 μg/ml von Willebrand factor (VWF) (Haemate P, CSL Behring, King of Prussia, PA) and 1.4 mg/ml ristocetin (ABP, Surrey, UK). Cells were then placed on a rotary shaker for 20 min at 360rpm and analysed on an Eclipse TE-200 inverted fluorescence microscope (Nikon, Tokyo, Japan) coupled to an Orca R2 CCD camera (Hamamatsu Photonics, Hamamatsu, Japan). For each condition, 4 experiments were conducted for all of which 3 contiguous pictures were taken and analysed using HC Image software (Hamamatsu Photonics). In all experiments, cell aggregates were identified using HC Image software by excluding single cells, doublets and triplets, and validated manually to remove eventual false aggregates (e.g. dust, debris). The number of aggregates and the surface area covered by each aggregate were then calculated using HC Image software.

### DAMI immunolabelling

For each condition, 1x10^6^ DAMI cells were transfected with pCMV-mi*GPIBA*-2+3-eGFP, pCMV-eGFP or with mock control differentiation medium using the Amaxa Nucleofector II and Cell Line Nucleofector Kit C (both Lonza, Basel, Switzerland) according to the manufacturer’s instructions. Cells were cultured in differentiation medium for 48h, after which GPIbα was detected using flow cytometry as described above. In parallel, immediately after nucleofection 1x10^5^ cells were transferred to a Lab-Tek chamber (Thermo Fisher Scientific, Waltham, MA) containing 1ml differentiation medium to examine the effects of GPIbα knockdown on cell morphology. After 48h, cells were fixed in 4% paraformaldehyde and stained with anti-GPIbα moAb 6B4 (20 μg/ml final) and a rabbit anti-mouse-FITC secondary Ab (30 μg/ml final) (Jackson Immunoresearch). The cytoskeleton was visualized by incubating cells with phalloidine-TRITC (7.5 μg/ml final) (Merck Millipore, Billerica, MA) and cells were mounted using ProLong Gold Antifade reagent containing DAPI (Life Technologies) for nuclear counterstaining.

Cells were analysed using a Nikon C1 confocal laser scanning microscope (Nikon) equipped with a Plan Apo VC 60× 1.4 NA oil immersion objective lens. Pictures were captured sequentially to prevent bleaching using appropriate excitation and emission filters for each fluorophore (DAPI—405 nm Argon laser with 450/50 nm band pass filter; FITC– 488 nm Helium-Neon laser with 525/50nm band pass filter and TRITC- 561nm Helium-Neon laser with a 605/40nm band pass filter). Image analysis was performed using EZ-C1 software (Nikon). Maximal length and width of the cells were determined by drawing a straight line on the confocal images along the cell axis and another one perpendicular to it, respectively. The cell aspect ratio was calculated by dividing the maximal width by the maximal length of each cell (n = 3 experiments; 3 replicates per experiment).

### Statistical analysis

All statistical analyses were performed using GraphPad Prism 5 (Graphpad Software, San Diego, CA). All data were analyzed by unpaired Student *t* test or one-way analysis of variance (ANOVA) followed by Dunnett’s or Tukey’s post-tests. Differences were considered statistically significant when * p ≤ 0.05, ** p ≤ 0.01 and *** p ≤ 0.001.

## Results

### *In silico* and *in vitro* siRNA selection and testing

**Fig 1:**
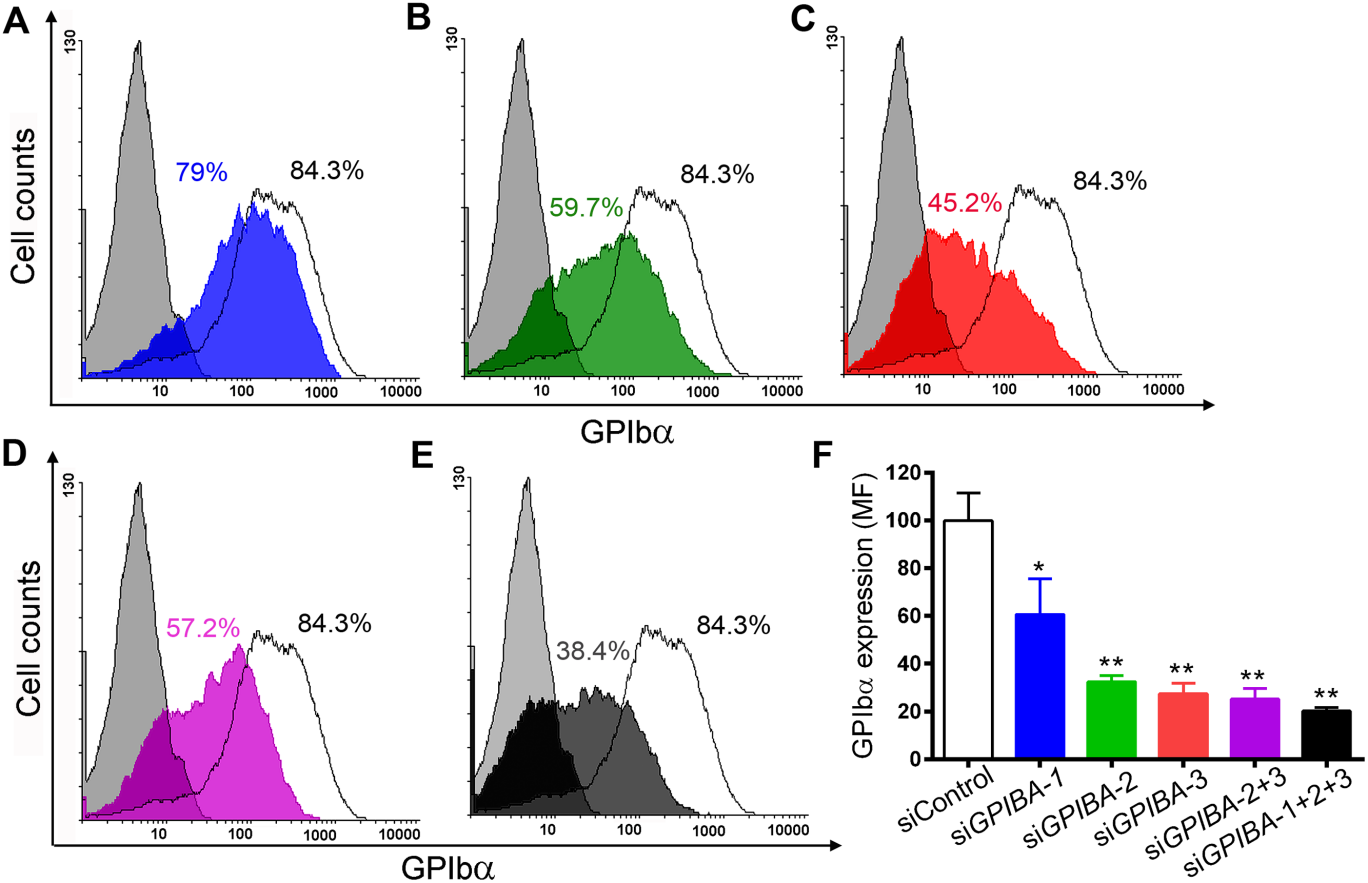
Knockdown of GPIbα expression following transfection of CHO GPIb-IX cells with siRNA. Representative flow cytometry histograms from mock transfected cells (white) and cells transfected with (A) si*GPIBA*-1 (blue), (B) si*GPIBA*-2 (green), (C) si*GPIBA*-3 (red), (D) si*GPIBA*-2+3 (purple) and (E) si*GPIBA*-1+2+3 (black) expressing GPIbα 48h post transfection. Negative control in which no anti-GPIbα moAb 6B4 was added is depicted in each histogram in grey. Percentages of cells expressing GPIbα are indicated next to each histogram. (F) Flow cytometry analysis representing mean fluorescence intensities of each population of CHO GPIb-IX cells expressing GPIbα ± SEM (n>3). Statistical analysis was performed using Anova followed by Dunnett’s post-test (* p<0.05; ** p<0.01).

As described under Materials and Methods, three siRNA sequences targeting different regions of *GPIBA* mRNA were selected and their ability to mediate GPIbα knockdown was evaluated 48h after transfection of CHO GPIb-IX cells with the siRNA duplexes. Transfection efficiencies using Oligo Block iT were around 45–50% 48h post transfection and were not significantly different between the experimental days (S1 Fig). A significant knockdown could be detected in CHO GPIb-IX cells transfected with si*GPIBA*-2, si*GPIBA*-3 or in combination of the two: 65.4 ± 5.7%, 72.9 ± 8.5% and 74.7 ± 4.5%, respectively (Fig 1B, 1C, 1D and 1F; p<0.001). Although si*GPIBA*-1 significantly downregulated GPIbα expression, it was not as efficient as the other two siRNAs (44.3 ± 0.2% knockdown; p<0.05) (Fig 1A and 1F). As expected, using si*GPIBA*-1 in combination with si*GPIBA*-2 and si*GPIBA*-3 did not significantly further increase the knockdown of GPIbα expression (74.7 ± 4.5% versus 79.7 ± 1.5%) (Fig 1E and 1F). Based on these results, si*GPIBA*-2 and si*GPIBA*-3 were selected for the development of miRNAs.

### Silencing of GPIbα by miRNA vectors

We developed miRNA vectors by omitting three nucleotides from si*GPIBA*-2 and si*GPIBA*-3 to generate mi*GPIBA*-2 and mi*GPIBA*-3 which were integrated in a human miR30 loop and flanking sequences, creating pre-miRNAs (pre-mi*GPIBA*-2 and pre-mi*GPIBA*-3) which were ultimately cloned into the pCMV-eGFP backbone in various configurations (S1 File, Table 1). When analyzing the transfected cells for GFP expression, a dramatic decrease in expression levels was observed in all CHO GPIb-IX cells transfected with miRNA vectors (Figure A in S2 File) as previously observed [23, 25, 26]. A loss of GFP expression was also observed when the miRNA sequence was placed at the C-terminal of the reporter gene. Indeed, CHO GPIb-IX cells transfected with pCMV-eGFP-mi*GPIBA*-2 exhibited a dramatic decrease in GFP expression compared to CHO GPIb-IX cells transfected with pCMV-eGFP (Figure A in S2 File). In light of these results, transfection efficiencies were determined using CHO GPIb-IX cells transfected with pCMV-eGFP in parallel with the *GPIBA* miRNA vectors and were routinely around 50% (Figure B in S2 File).

**Fig 2:**
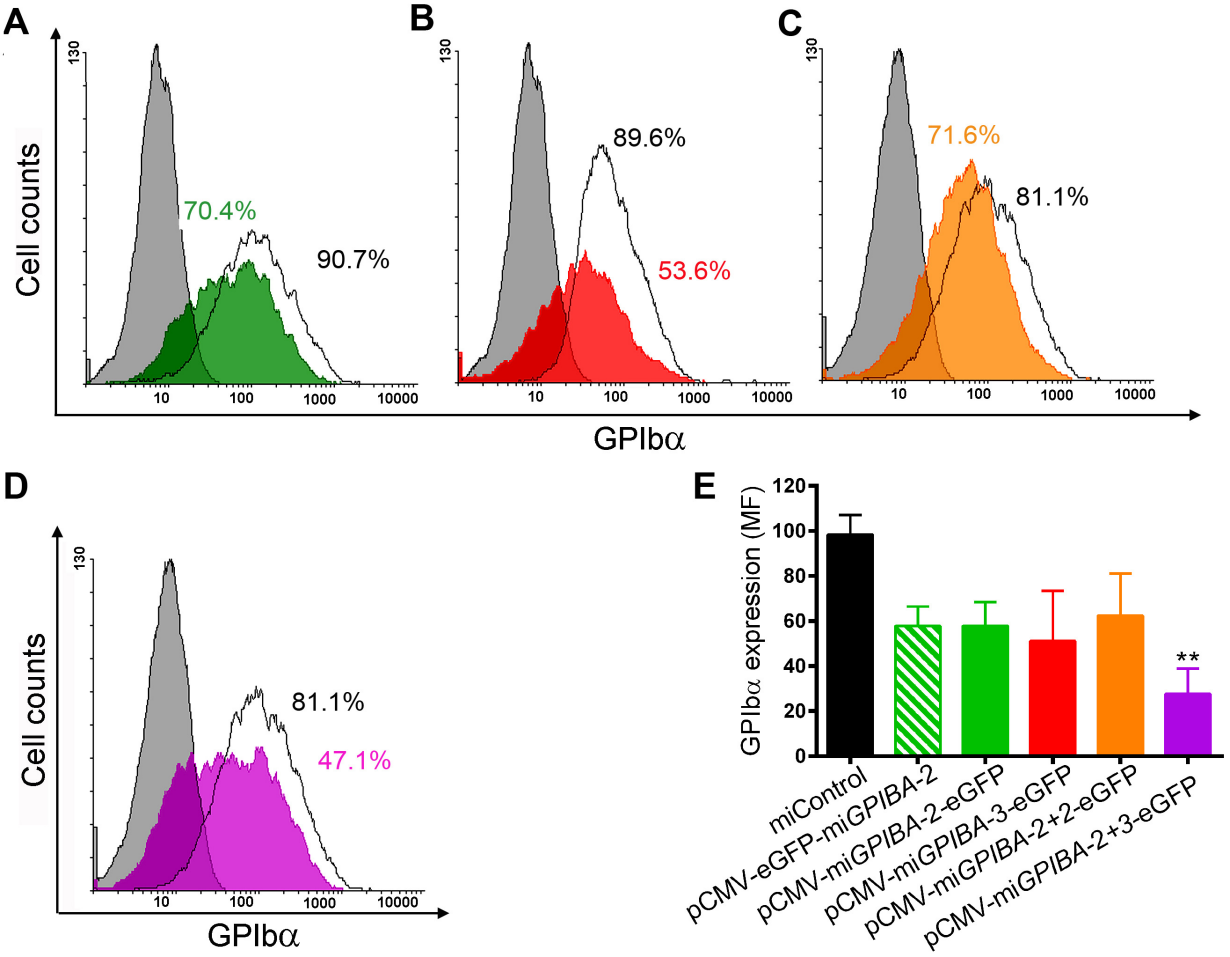
miRNA-mediated knockdown of GPIbα by transfection of CHO GPIb-IX cells. Representative flow cytometry histograms from CHO GPIb-IX control cells (white) and cells transfected with (A) pCMV-mi*GPIBA*-2-eGFP (green), (B) pCMV-mi*GPIBA*-3-eGFP (red), (C) pCMV-mi*GPIBA*-2+2-eGFP (orange), or (D) pCMV-mi*GPIBA*-2+3-eGFP (purple) expressing GPIbα 48h post transfection. Negative control in which no anti-GPIbα moAb 6B4 was added is depicted in each histogram in grey. Percentages of cells expressing GPIbα are indicated next to each histogram. (F) Flow cytometry analysis representing mean fluorescence intensities of each population of CHO GPIb-IX cells expressing GPIbα ± SEM (n>3). Statistical analysis was performed using Anova followed by Dunnett’s post-test (** p<0.01).

Although transfection with pCMV-mi*GPIBA*-2-eGFP and pCMV-mi*GPIBA*-3-eGFP resulted in reduced GPIbα expression (41.1 ± 10.7% and 48.1 ± 22.6% knockdown, respectively) (Fig 2A, 2B and 2E), it did not reach statistical significance. Similar results were also obtained with pCMV-eGFP-mi*GPIBA*-2 (Fig 2E). Since mammalian miRNAs function mainly by mRNA degradation for which binding of the central nucleotide 10–14 is critical, we designed non-specific miRNAs by mutating this region of the sense and antisense sequence of each miRNA to their reverse complement, thus generating pCMV-mi*GPIBA*-2NS-eGFP and pCMV-mi*GPIBA*-3NS-eGFP (S1 File) [22, 23]. Transfection of CHO GPIb-IX cells with these constructs indeed did not affect GPIbα expression thus confirming the specificity of mi*GPIBA*-2 and mi*GPIBA*-3 (S2 Fig and data not shown).

In order to improve knockdown of GPIbα, we combined multiple miRNAs in a single plasmid as this approach has been reported to be successful (Figure B in S1 File) [26]. Remarkably, expression of GPIbα in CHO GPIb-IX cells transfected with the pCMV-mi*GPIBA*-2+2-eGFP containing two identical miRNA sequences was not significantly decreased compared to control CHO GPIb-IX cells or cells transfected with a miRNA vector containing mi*GPIBA*-2 or mi*GPIBA*-3 (38.6 ± 17.5% knockdown; p>0.05; Fig 2C and 2E). However, when using a combination of two different miRNA sequences, a significantly higher knockdown of GPIbα expression could be achieved compared to either individual miRNAs (71.9 ± 6.6%; p ≤ 0.01) (Fig 2D and 2E).

### GPIbα downregulation reduces ristocetin-induced von Willebrand factor-GPIb dependent cell aggregation

**Fig 3:**
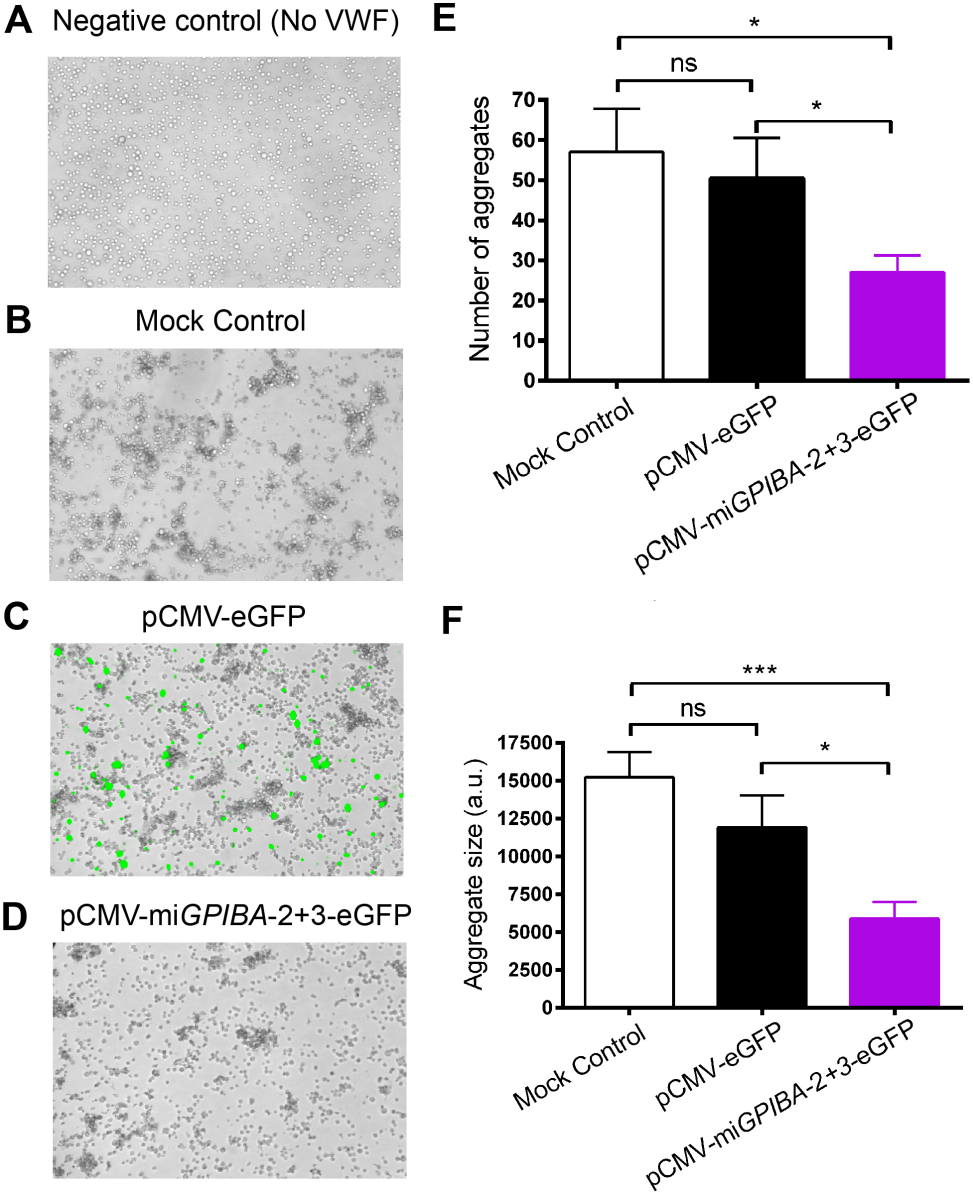
Knockdown of GPIb-IX reduces ristocetin induced VWF-dependent CHO GPIb-IX cell aggregation. CHO GPIb-IX cells were incubated with ristocetin without (A) or with (B-E) VWF on a rotary shaker to induce aggregate formation. Representative overlay bright field and fluorescent (GFP) images from (A-B) mock, (C) pCMV-eGFP and (D) pCMV-mi*GPIBA*-2+3-eGFP transfected cells are shown. Quantitative analysis was performed by measuring (E) the number of aggregates and (F) the aggregate size (a.u.: arbitrary units). Data represent mean ± SEM (n = 4). Statistical analysis was performed using the unpaired Student t test (* p<0.05).

Since successful silencing of GPIbα should impair the interaction with its main ligand VWF, we performed cell aggregation assays with mock, pCMV-eGFP or pCMV-mi*GPIBA*-2+3-eGFP transfected CHO GPIb-IX cells in the presence of VWF and ristocetin, which is needed to induce the interaction between GPIbα and VWF in this type of assay. Following rotary shaking, the number and size of aggregates formed were evaluated and was similar in pCMV-eGFP or mock transfected CHO GPIb-IX cells (50.6 ± 9.9 vs. 57.0 ± 10.8 aggregates and 15.2 ± 1.6 vs. 11.9 ± 2.1 arbitrary units (A.U.), respectively) (Fig 3B, 3C, 3E and 3F). As expected, no aggregate could be formed when VWF was omitted in the assay (Fig 3A). Transfection of CHO GPIb-IX cells with pCMV-mi*GPIBA*-2+3-eGFP exhibited reduced number (27.1 ± 4.1; p<0.05) and size of the aggregates (5.1 ± 1.1 A.U., p ≤ 0.01) (Fig 3D–3F). Further analysis revealed that the average aggregate size was 32.7 ± 8.6% smaller in mean aggregate size, despite the fact that only 15.4 ± 1.3% of the cells were successfully transfected and thus lacking GPIbα expression. This prompted us to perform a control experiment in which CHO GPIb-IX cells were mixed with CHO β9 cells in a 85:15 ratio before performing the aggregation assay, thus mimicking the *GPIBA* miRNA transfection conditions. Under these conditions, a reduction in the number of aggregates (33.4 ± 1.6%) and mean aggregate size (27.0 ± 1.5%) was observed as compared to a population of 100% CHO GPIb-IX cells, thus validating our results observed in CHO GPIb-IX cells transfected with pCMV-mi*GPIBA*-2+3-eGFP (S3 File).

### Down regulation of GPIbα expression in megakaryoblastic DAMI cells triggers reorganization of the actin network

**Fig 4:**
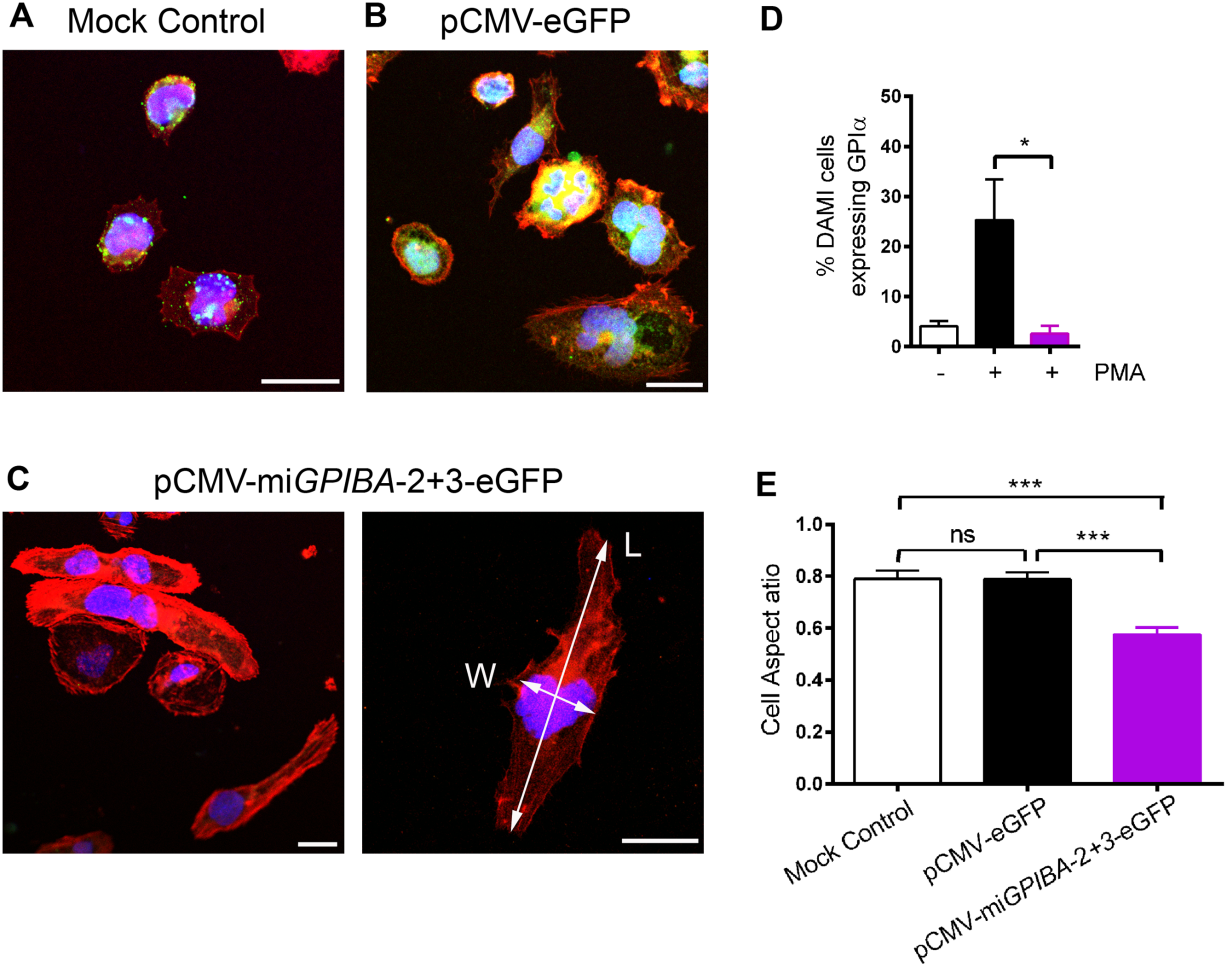
Morphological changes in differentiating megakaryoblastic DAMI cells. (A-C) Representative confocal images of differentiating mock transfected DAMI cells (A), DAMI cells transfected with pCMV-eGFP (note extensive eGFP fluorescence) (B) and DAMI cells transfected with pCMV-mi*GPIBA*-2+3-eGFP (C-D). Cells were stained for GPIbα (green), the actin cytoskeleton (red) and the nucleus (blue/purple). Scale bar is 25 μm. The width (W) and length (L) of the cells along two perpendicular axes used to calculate cell aspect ratios are indicated in (C). (D) GPIbα expression in untransfected (white) or pCMV-eGFP (black) or pCMV-mi*GPIBA*-2+3-eGFP (purple) transfected DAMI cells was determined by flow cytometry. DAMI transfected cells were stimulated with PMA for 48h as indicated. Data represent mean fluorescence intensities of GPIbα expression ± SEM (n>3). (E) Quantitative analysis showing cell aspect ratios (W/L) represent mean ± SEM (n >3; 20 cells analyzed per condition). Statistical analysis was performed using the unpaired Student t test (* p<0.05; *** p<0.01).

Another hallmark of GPIbα dysfunction or deficiency is abnormal megakaryopoiesis and the formation of giant platelets. Moreover, the validation of the miRNA constructs in human megakaryocytic or megakaryoblastic cell types is an important milestone towards the ultimate goal of achieving stable RNAi-mediated target gene knockdown in CD34^+^ HSPC. We therefore studied the effects of GPIbα deficiency during megakaryopoiesis by knocking down GPIbα expression in megakaryoblastic DAMI cells. A subset of the DAMI cell population expresses the MK- and platelet-markers GPIb-V-IX and αIIbβ3. Surface expression of both receptors can be upregulated by stimulating differentiation of the cells to a more mature phenotype [29]. In our hands, PMA-induced differentiation led to shape change and adhesion of cells concomitant with an increase in ploidy (from predominantly 2N to 4N and going up to 16N) and an upregulation of both GPIbα (from 4.1 ± 1.0% to 25.3 ± 8.1%; Fig 4D) and αIIbβ3 (from 11.9 ± 0.7% to 73.2 ± 7.0%; n = 3) (data not shown). To validate the use of miRNA vectors in MK/platelet lineage cells, DAMI cells were nucleofected with pCMV-mi*GPIBA*-2+3-eGFP, and stimulated with PMA to induce GPIbα expression. After 2 days, 64.4 ± 6.5% of eGFP-nucleofected DAMI cells expressed eGFP. In contrast to mock- or eGFP-nucleofected DAMI cells, no upregulation of GPIbα expression could be detected in pCMV-mi*GPIBA*-2+3-eGFP nucleofected DAMI cells and GPIbα expression remained at baseline expression levels (2.6 ± 0.8%; p<0.05), attesting of a successful knockdown of GPIbα. In addition, overt differences in actin organization could be observed in *GPIBA* miRNA-transfected cells compared to mock- or eGFP-transfected cells, with *GPIBA* miRNA-transfected DAMI cells displaying a more stretched or elongated morphology (Fig 4A–4C). These morphological cell shape changes could be quantified by calculating the cell aspect ratio which gives an estimate of overall cell morphology. Cells lacking detectable GPIbα showed a significantly lower cell aspect ratio compared to mock- and eGFP-transfected cells (0.57 ± 0.03 vs. 0.79 ± 0.03 and 0.78 ± 0.03 respectively; p ≤ 0.001) (Fig 4E), confirming that miRNA technology can be successfully used to study the function of a candidate gene during megakaryopoiesis.

## Discussion

Over the last decade, technological advances with genomics, transcriptomics or proteomics have revolutionised human genetic research including our knowledge in platelet biology. Nevertheless, it remains a challenge to assign function to thousands of candidate genes/proteins in platelets as direct molecular biology approaches cannot be applied due to their anucleated nature. We here report on the development of a tool using miRNA vectors to successfully knockdown a platelet protein GPIbα in a megakaryoblastic cell line that could be applied to HSPC in view to generate genetically modified human platelets.

We started developing our strategy by first selecting different siRNAs based on multiple parameters defining an efficient siRNA capable of successfully downregulating a target gene. All selected siRNAs were successful at downregulating GPIbα expression in CHO GPIb-IX cells, albeit to a different extent, with si*GPIBA*-2 and mi*GPIBA*-3 being the most powerful (Fig 1). These findings corroborate the general recommendation that despite extensive *in silico* screening procedures and the publication of numerous siRNA design guidelines, only actual *in vitro* or *in vivo* tests provides truly reliable information regarding the knockdown potential of a siRNA [20, 21].

Although the use of siRNAs allows rapid screening for efficient target sequences, direct introduction of siRNA in platelets is currently hampered by a low transfection efficiency [5]. Moreover, *de novo* synthesis of proteins in anucleate platelets only occurs from a limited number of cytoplasmic mRNAs [30] and most platelet proteins are synthesized in the MK from which platelets originate [6]. Hence, the best method to obtain and study platelets or MK lacking expression of one or more proteins is by genetic modification of nucleated platelet progenitor cells. We therefore converted siRNAs to plasmid DNA-based miRNAs, which can be transferred to vectors capable of integrating in the host genome, thus resulting in stable and long term knockdown [31, 32].

A report from Amendola *et al*. suggests that the use of a scaffold derived from an endogenous miRNA with abundant expression in the target cell type, results in a high silencing efficiency [31]. Therefore, we opted to integrate the siRNA sequences in human miR30 loop and flanking regions, since the presence of the endogenous human miR30 in human platelets has been confirmed using microarray technology [33]. We used a miRNA vector where miRNA sequences could be inserted in the 3’ or 5’ of a reporter gene (eGFP), however observed that no GFP fluorescence could be detected in either CHO GPIb-IX or DAMI cells transfected with mi*GPIBA* constructs regardless of the 3’ or 5’ position of the reporter gene (Figure A in S2 File). This is in agreement with the literature [25, 26] where the hairpin loop structure of miRNAs reduces the translation efficiency of the reporter gene. Indeed, since the initial phase of our study, this loss of reporter expression has been circumvented by the addition of a chimeric intron into the construct [24, 25, 31]. In addition, other scaffold for miRNA constructs such as miR155, miR223 or miR451 have been used as well, the latter showing improved Ago2 specificity and reducing risk of overwhelming the endogenous miRNA machinery [24, 31, 34].

Although in all single miRNA constructs tested the observed knockdown was much weaker compared to the corresponding siRNAs, we could reach similar efficacies by inserting additional miRNA hairpins, thereby providing a certain level of control over the knockdown obtained (Figs 1 and 2). This can be particularly useful when dealing with a highly expressed target protein, such as the GPIb-V-IX complex in MK and platelets, where silencing using a single miRNA might be ineffective because of the so-called dilution effect [35]. In order to increase GPIbα knockdown, we initially inserted two identical miRNA hairpins in one plasmid, a strategy successfully adopted in other cell types [26, 31, 36]. While no further reduction in GPIbα expression could be achieved using two identical miRNA sequences, a combination of two different miRNAs (pCMV-mi*GPIBA*-2+3-eGFP) did result in increased GPIbα silencing (Fig 2). These results partially confirm those of Amendola *et al*. who showed that a tandem configuration of two completely identical miRNAs is unstable, in contrast to a configuration of two different miRNAs [31]. However, in our case both miRNAs have an identical human miR30 loop and flanking sequences and only differ in their targeting sequence, suggesting that different targeting sequences might be sufficient to circumvent structural tandem (artificial) miRNA instability.

In order to validate miRNA technology for functional studies, we sought to mimic the effects of GPIbα deficiency on platelet aggregation by performing a CHO GPIb-IX cell aggregation assay which is dependent on the interaction between GPIbα and its main ligand VWF [18, 28]. Transfection of CHO GPIb-IX cells with pCMV-mi*GPIBA*-2+3-eGFP significantly reduced the number of aggregates formed (Fig 3E), as well as the aggregate size (Fig 3F), illustrating that miRNA technology can be successfully used for functional studies at the receptor-ligand level.

To further demonstrate the potential of the miRNA-based approach for gene silencing during megakaryopoiesis, the mi*GPIBA*-2+3 cassettes were introduced into megakaryoblastic DAMI cells to evaluate the effects of downregulating GPIbα on actin reorganisation during PMA-induced differentiation. When GPIbα upregulation was blocked in differentiating DAMI cells, we observed a different actin distribution with an elongated cell morphology represented by a significantly reduced cell aspect ratio compared to mock- and eGFP-transfected control conditions. This is in line with the role of GPIbα in actin reorganisation via the binding of its intracellular tail domain to actin-binding proteins such as Filamin [37, 38]. Indeed, both mice lacking Filamin A or GPIbα are unable to anchor the plasma membrane to the cytoskeleton and have enlarged platelets [39–41].

In conclusion, we show that our miRNA-based approach is a powerful tool to successfully silence a platelet gene and can also be used for functional studies. During the preparation of this manuscript, few examples using miRNA backbone vectors have been applied to silence a platelet gene in human HSPC with success [38, 42]. We therefore believe that this miRNA strategy could be of great use in the characterisation of recently discovered platelet genes with unknown function, thereby identifying potential new targets for the development of novel antithrombotics but also for other applications such as engineering HLA-universal platelets [16].

## Supporting Information

S1 Fig Transfection efficiencies of siRNA oligonucleotides in CHO GPIb-IX cells. Transfection efficiencies assessed by % of CHO GPIb-IX cells expressing GFP ± SEM (n>3), 48h post transfection with OligoBlock iT carried out in parallel with si*GPIBA*-1 (blue), si*GPIBA*-2 (green), si*GPIBA*-3 (red), si*GPIBA*-2+3 (purple) or si*GPIBA*-1+2+3 (black) transfections. Note that the transfection efficiencies are not significantly different between the groups. Statistical analysis was performed using Anova followed by Tukey’s post-test (p>0.05).
(TIF)

S2 Fig Transfection of CHO GPIb-IX cells with pCMV-miGPIBA-2NS-eGFP does not alter GPIbα expression. Representative flow cytometry histogram from CHO GPIb-IX control cells (grey area) and cells transfected with pCMV-mi*GPIBA*-2NS-eGFP (black line) expressing GPIbα 48h post transfection. Negative control in which no anti-GPIbα moAb 6B4 was added is depicted by a black line with white area.
(TIF)

S1 File Pre-amiR structures and plasmid constructs. (A) Predicted stem-loop hairpin structures for mi*GPIBA*-2 and mi*GPIBA*-3. Structures were predicted using RNA Structure software V5.1. (http://rna.urmc.rochester.edu/RNAstructure.html) (B) Schematic representation of the miRNA constructs tested. CMV: CMV promoter, eGFP: eGFP coding sequence, green hairpin: mi*GPIBA*-2, blue hairpin: mi*GPIBA*-3 red hairpin mi*GPIBA*-2NS, grey hairpin: mi*GPIBA*-3NS.
(TIF)

S2 File Transfection efficiencies of miRNA constructs in CHO GPIb-IX cells. (A) Flow cytometric analysis representing mean fluorescence intensities (MF) ± SEM (n>3) of GFP expression in CHO GPIb-IX cells transfected with pCMV-eGFP (black), pCMV-eGFP-mi*GPIBA*-2 (hatched green), pCMV-mi*GPIBA*-2-eGFP (green), pCMV-mi*GPIBA*-2+2-eGFP (orange), or pCMV-mi*GPIBA*-2+3-eGFP (purple). Note high expression for pCMV-eGFP transfected cells and loss of GFP expression for cells transfected with miRNA constructs. Statistical analysis was performed using Anova followed by Dunnett’s post-test (** p<0.01). (B) Transfection efficiencies assessed by % of CHO GPIb-IX cells expressing GFP ± SEM (n>3), 48h post transfection with pCMV-eGFP carried out in parallel with pCMV-eGFP-mi*GPIBA*-2 (hatched green), pCMV-mi*GPIBA*-2-eGFP (green), pCMV-mi*GPIBA*-2+2-eGFP (orange), or pCMV-mi*GPIBA*-2+3-eGFP (purple) transfections. Note that the transfection efficiencies are not significantly different between the groups. Statistical analysis was performed using Anova followed by Tukey’s post-test (p>0.05).
(TIF)

S3 File Ristocetin induced VWF-dependent cell aggregation. CHO GPIb-IX cells were incubated with ristocetin and VWF on a rotary shaker to induce aggregate formation. (A-C) Representative pictures from 100% CHO GPIb-IX cells (A), 100% CHO β9 cells (B) and a mixture of 85% CHO GPIb-IX cells and 15% CHO β9 cells (C) are shown. Scale bar is 100μm. Quantitative analysis was performed by measuring the number of aggregates (D) and the aggregate size (a.u.: arbitrary units) (E). Data represent mean ± SEM (n = 6). Statistical analysis was performed using the unpaired Student t test (*** p<0.001).
(TIF)
