# Supplementary figures and images for: Artificial MiRNA Knockdown of Platelet Glycoprotein lbα: A Tool for Platelet Gene Silencing
Source: PLoS One. 2015 Jul 15;10(7):e0132899. doi: 10.1371/journal.pone.0132899 (PMC4503784; doi:10.1371/journal.pone.0132899)

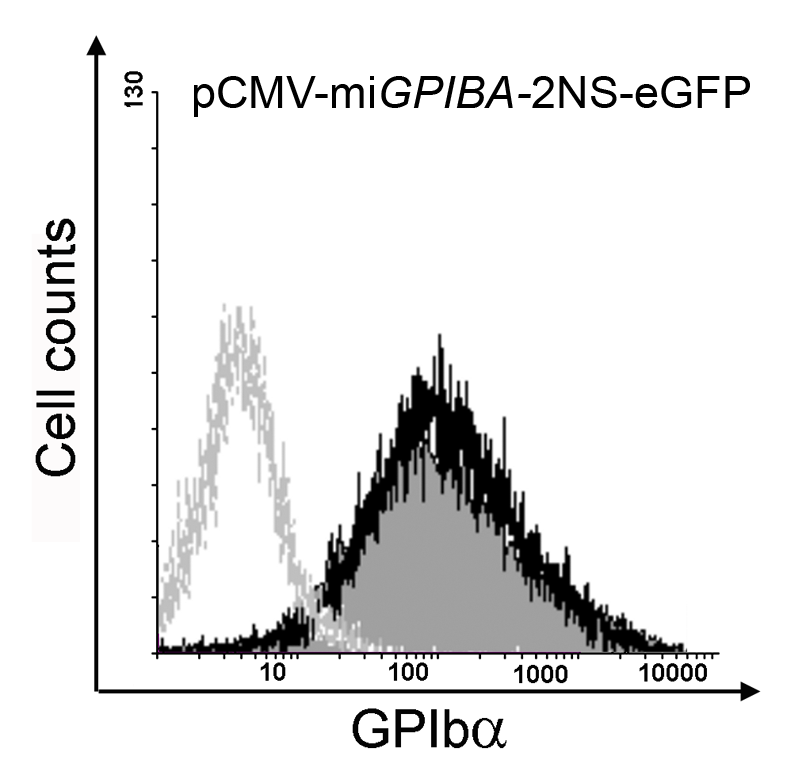

Supplement: S2 Fig — Representative flow cytometry histogram from CHO GPIb-IX control cells (grey area) and cells transfected with pCMV-miGPIBA-2NS-eGFP (black line) expressing GPIbα 48h post transfection. Negative control in which no anti-GPIbα moAb 6B4 was added is depicted by a black line with white area. (TIF) [file pone.0132899.s002.tif]

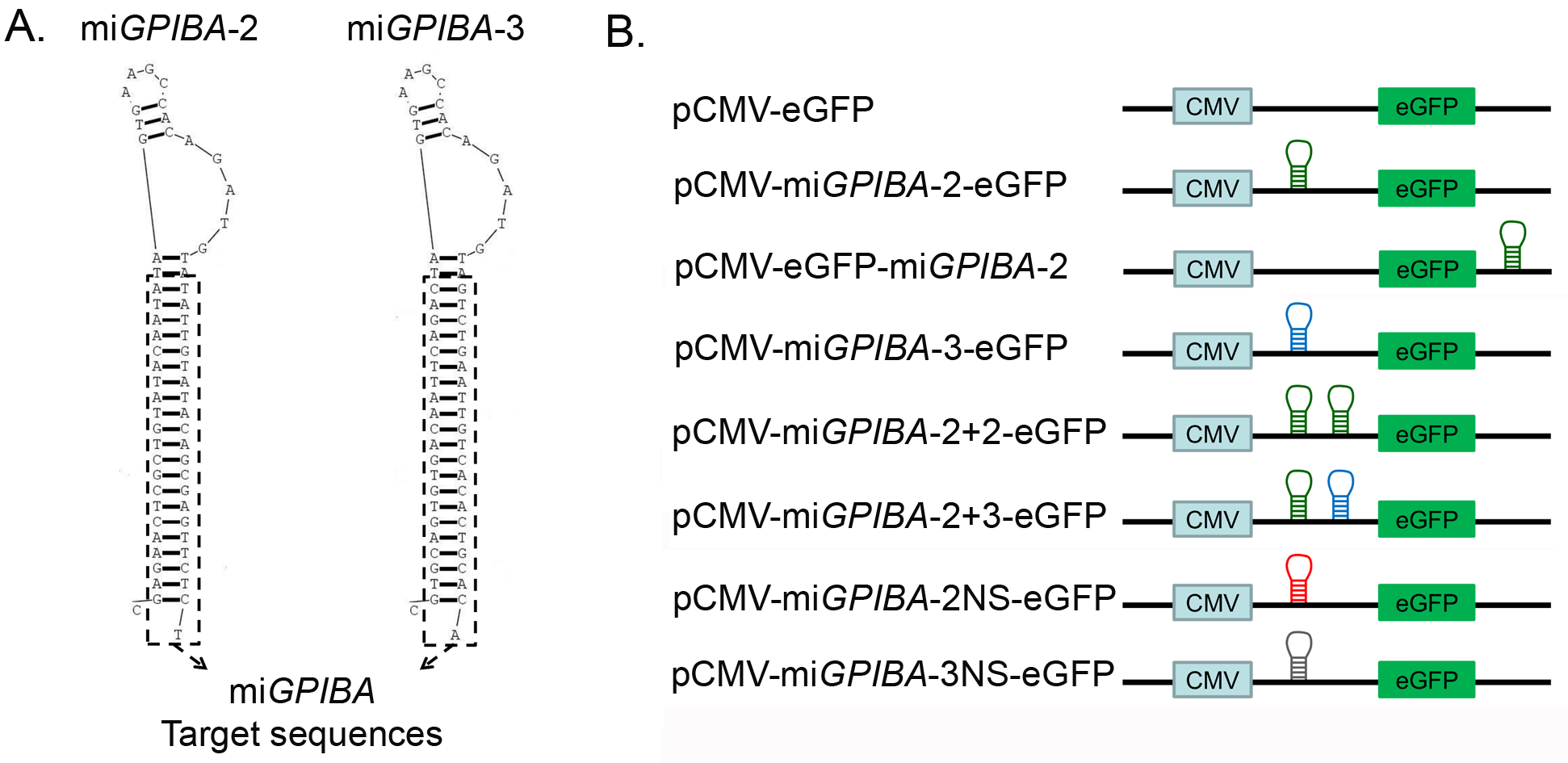

Supplement: S1 File — (A) Predicted stem-loop hairpin structures for miGPIBA-2 and miGPIBA-3. Structures were predicted using RNA Structure software V5.1. (http://rna.urmc.rochester.edu/RNAstructure.html) (B) Schematic representation of the miRNA constructs tested. CMV: CMV promoter, eGFP: eGFP coding sequence, green hairpin: miGPIBA-2, blue hairpin: miGPIBA-3 red hairpin miGPIBA-2NS, grey hairpin: miGPIBA-3NS. (TIF) [file pone.0132899.s003.tif]

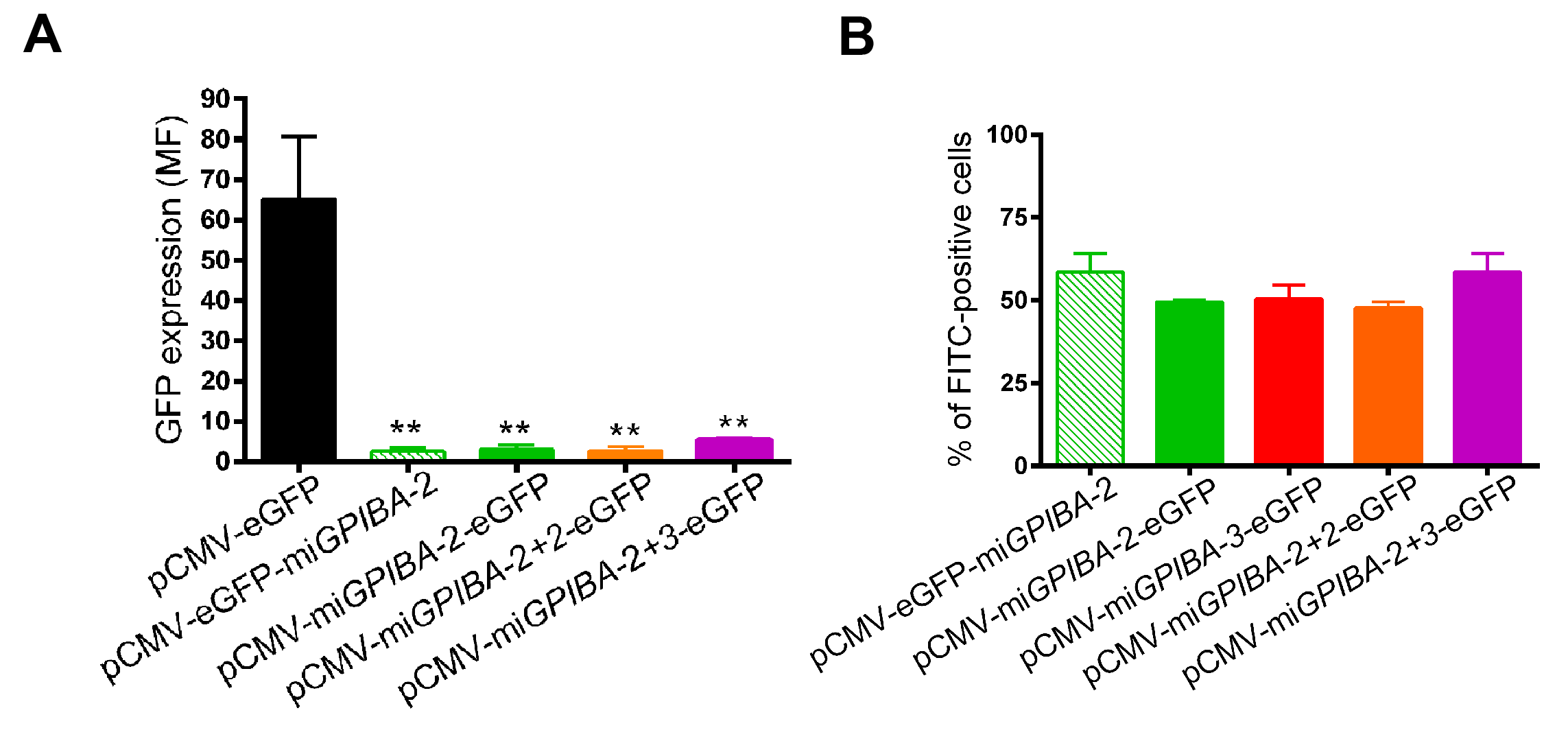

Supplement: S2 File — (A) Flow cytometric analysis representing mean fluorescence intensities (MF) ± SEM (n>3) of GFP expression in CHO GPIb-IX cells transfected with pCMV-eGFP (black), pCMV-eGFP-miGPIBA-2 (hatched green), pCMV-miGPIBA-2-eGFP (green), pCMV-miGPIBA-2+2-eGFP (orange), or pCMV-miGPIBA-2+3-eGFP (purple). Note high expression for pCMV-eGFP transfected cells and loss of GFP expression for cells transfected with miRNA constructs. Statistical analysis was performed using Anova followed by Dunnett’s post-test (** p<0.01). (B) Transfection efficiencies assessed by % of CHO GPIb-IX cells expressing GFP ± SEM (n>3), 48h post transfection with pCMV-eGFP carried out in parallel with pCMV-eGFP-miGPIBA-2 (hatched green), pCMV-miGPIBA-2-eGFP (green), pCMV-miGPIBA-2+2-eGFP (orange), or pCMV-miGPIBA-2+3-eGFP (purple) transfections. Note that the transfection efficiencies are not significantly different between the groups. Statistical analysis was performed using Anova followed by Tukey’s post-test (p>0.05). (TIF) [file pone.0132899.s004.tif]

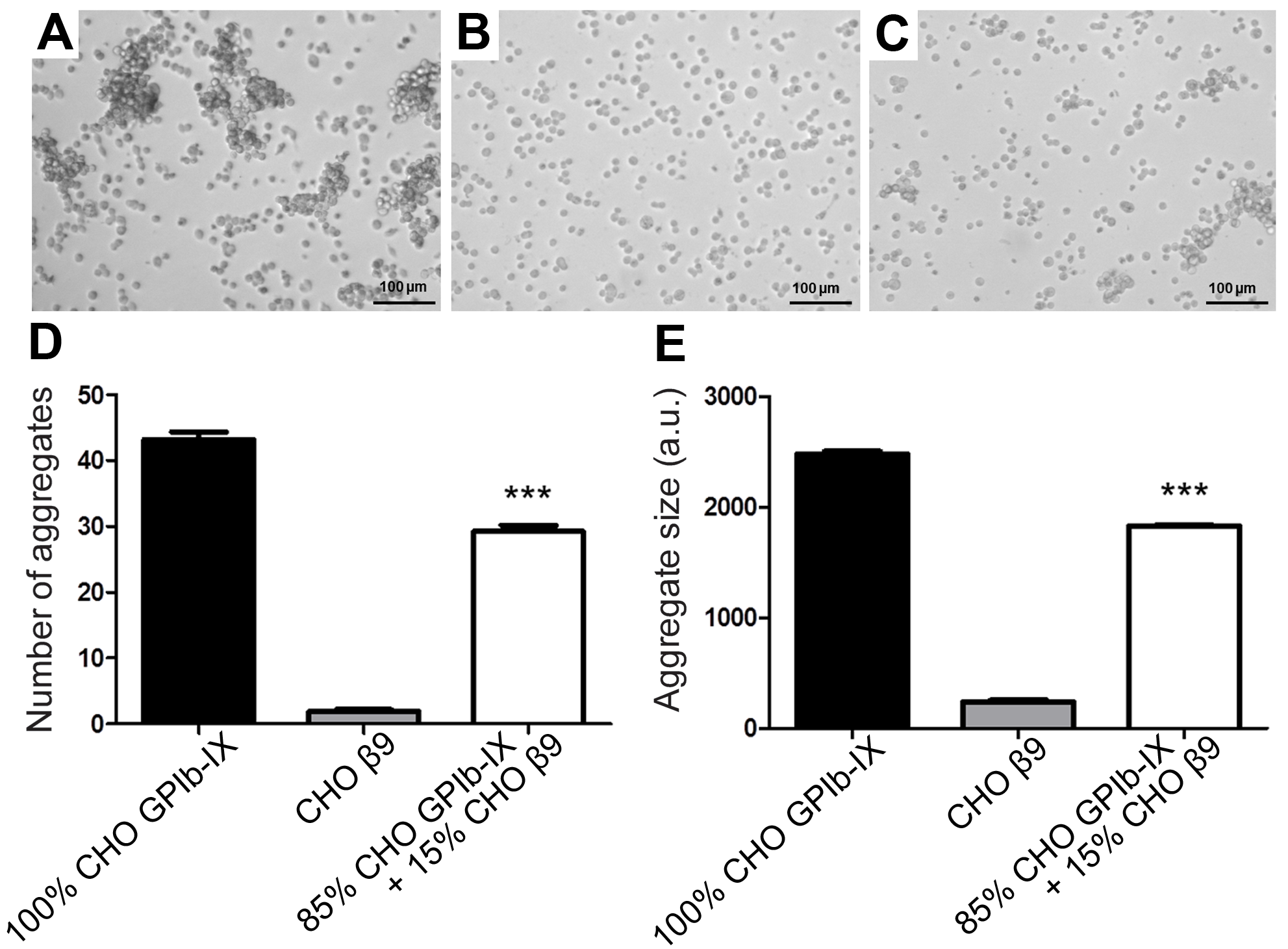

Supplement: S3 File — CHO GPIb-IX cells were incubated with ristocetin and VWF on a rotary shaker to induce aggregate formation. (A-C) Representative pictures from 100% CHO GPIb-IX cells (A), 100% CHO β9 cells (B) and a mixture of 85% CHO GPIb-IX cells and 15% CHO β9 cells (C) are shown. Scale bar is 100μm. Quantitative analysis was performed by measuring the number of aggregates (D) and the aggregate size (a.u.: arbitrary units) (E). Data represent mean ± SEM (n = 6). Statistical analysis was performed using the unpaired Student t test (*** p<0.001). (TIF) [file pone.0132899.s005.tif]
